# Proteomic analysis of differential biological responses and fatty acid metabolism in slow-growing chickens fed dietary tuna oil directly or via glucose transporter-targeted nanoparticles

**DOI:** 10.1016/j.psj.2026.106405

**Published:** 2026-01-07

**Authors:** Piyaradtana Homyok, Pramin Kaewsatuan, Valérie Labas, Daniel Tomas, Ana Paula Teixeira-Gomes, Elisabeth Baéza, Cécile Berri, Amonrat Molee, Wittawat Molee

**Affiliations:** a School of Animal Technology and Innovation, Institute of Agricultural Technology, Suranaree University of Technology, Nakhon Ratchasima 30000, Thailand; b INRAE, CNRS, Université de Tours, UMR PRC, Nouzilly 37380, France; c INRAE, Université de Tours, CHU de Tours, Imaging Facility for Phenotyping from Animal to Molecule (PIXANIM), Nouzilly 37380, France; d INRAE, Université de Tours, UMR BOA, Nouzilly 37380, France

**Keywords:** Lipid-based nanoparticles, n-3 fatty acids, Proteomic, Slow-growing chicken, Tuna oil

## Abstract

This study investigated the effects of tuna oil **(PC)** and tuna oil encapsulated in lipid nanoparticles **(TNP)** on metabolic pathways in thigh muscle of slow-growing chickens using proteomic analysis. Compared with the negative control **(NC)** diet containing 6% rice bran oil, the PC and TNP diets replaced half of the rice bran oil with tuna oil, thereby increasing dietary n-3 PUFAs and altering the n-6/n-3 ratio. Iliotibialis muscle samples were collected from slow-growing Korat chickens (*n* = 6 for each PC and TNP; *n* = 5 for NC). Fatty acid profiles were analyzed by gas chromatography-mass spectrometry following lipid extraction and methylation. Proteomics analysis was performed using polyacrylamide gel-based protein separation and nanoLC-MS/MS to identify and quantify differentially expressed proteins. Distinct biological responses were observed between dietary treatments. In the TNP group, energy metabolism shifted toward aerobic pathways, with increased β-oxidation and reduced reliance on alternative pathways, such as the creatine-phosphate system, to support the tricarboxylic acid cycle. In contrast, the PC group exhibited high lipid peroxidation and by-products, triggering robust antioxidant and detoxification responses, as well as membrane repair mechanisms. Although the TNP group also activated antioxidant defenses, the response was less pronounced and was accompanied by increased expression of proteins involved in vesicle trafficking. Lipid peroxidation in the PC group was associated with calcium influx to maintain calcium homeostasis and stabilize muscle contraction under oxidative stress. This was evidenced by the upregulation of proteins related to sarcoplasmic reticulum calcium pumps and muscle contraction stabilization. Conversely, the TNP group demonstrated adaptive responses to increased contractile activity with lower oxidative burden. Regarding immune function, the PC group showed stronger MHC-based immunosurveillance, reflecting heightened oxidative stress. Although immune responses were less pronounced in the TNP group, immune surveillance was maintained through selective protein expression. Overall, these findings demonstrate distinct cellular strategies in response to oxidative stress and immune challenges between PC and TNP treatments, highlighting the potential of lipid nanoparticle systems to optimize dietary lipid delivery in poultry.

## Introduction

Omega-3 polyunsaturated fatty acids (**n-3 PUFAs**) are widely recognized for their health benefits, including cardiovascular protection and anti-inflammatory properties (Simopoulos, 2008; Calder, 2015). These benefits have prompted the incorporation of n-3 PUFAs into poultry diets as a strategy to enhance the nutritional value of meat, particularly in slow-growing chicken breeds (Hang et al., 2018; Khosinklang et al., 2023). However, challenges remain in maximizing the bioavailability and stability of these essential fatty acids, including the need to mitigate lipid oxidation during feed processing and improving their utilization in animal metabolism.

Lipid-based nanotechnology offers a promising solution for targeted delivery of n-3 PUFAs, addressing these challenges. Nanoparticles protect n-3 PUFAs from oxidative degradation (Chanburee and Tiyaboonchai, 2017; Chang and Nickerson, 2018) and enhance their absorption (Tian et al., 2017), facilitating their incorporation into animal tissues (Smith et al., 2020). These advancements could significantly improve the nutritional profile of meat products. Recent research has demonstrated that glucose transporter-targeted nanoparticles can be absorbed by intestinal epithelial cells in vitro (Wangngae et al., 2023), by C2C12 skeletal muscle cell (Yeh et al., 2014) and subsequently reach target tissues such as skeletal muscle in vivo (Homyok et al., 2025). These observations point to the potential of nanoencapsulation technologies to optimize n-3 PUFA delivery and functionality in meat production.

As reported in a previous study, supplementation with n-3 PUFA in slow-growing chickens modulates the expression of genes such as l-FABP, which plays a crucial role in regulating lipid metabolism in response to long-chain PUFAs (Khosinklang et al., 2023). Although these gene-level findings provide valuable insights, they do not fully reflect downstream effects at the protein level, which ultimately govern cellular function. Therefore, proteomic profiling is needed to characterize the protein networks and metabolic pathways underlying the observed phenotype. Furthermore, because the present study introduces a nanoparticle-based delivery approach in poultry, alterations may extend beyond lipid metabolism alone, underscoring the importance of protein-level analyses to comprehensively elucidate cellular responses to nanoparticle exposure. Despite these promising developments, comprehensive evaluations of nanoencapsulation technologies in agricultural contexts remain limited. There is a critical need to examine how these technologies influence biological processes, protein expression, and overall meat quality in livestock. Such insights are essential to bridge the gap between laboratory advancements and their practical applications in sustainable animal production systems (Jones and Ricke, 2021).

This study addresses this gap by comparing the effects of free (**PC**) and nanoencapsulated tuna oil (**TNP**) on fatty acid profiles and protein expression in the thigh muscle of slow-growing Korat chickens, a tissue with higher lipid content than breast muscle. The diets were formulated to evaluate the effects of n-3 PUFA supplementation. The negative control (**NC**) diet contained 6% rice bran oil, which is rich in n-6 PUFA. In contrast, the positive control (PC) and TNP diets replaced 50% of the rice bran oil with tuna oil, thereby increasing n-3 PUFA content and altering the n-6/n-3 ratio. In this context, we aimed to identify delivery-format-associated proteomic signatures that could clarify whether nanoencapsulation offers advantages for improving the functional quality of meat.

## Materials and methods

### Ethics statement

All procedures in the present study were approved by the Ethics Committee on Animal Use of the Suranaree University of Technology, Nakhon Ratchasima, Thailand (SUT-IACUC-002/2022).

### Birds and housing

One-day old female Korat chickens were reared on floor pens with a density of 8 birds/m^2^ until 49 d of age. The birds received feed and drinking water ad libitum. They were vaccinated against Marek’s disease at the hatchery, followed by vaccines against Newcastle disease and infectious bronchitis at 7 and 21 d of age. In addition, a vaccine against Gumboro disease was administered at 14 d of age. The chickens were reared together from hatching until 49 d of age, reaching an average body weight of 903.14 ± 17.71 g.

### Experiment chicken and tissue collection

The experimental model was a completely randomized design, which included three dietary treatments, 5 replications and twenty-five 49-d-old female chickens per replication. A basal diet was formulated based on corn-soybean containing 6% of rice bran oil as the negative control (NC), 3% of the rice bran oil content were replaced with 3% tuna oil as positive control (PC) or 3% tuna oil in targeted nanoparticles (TNP). The preparation of TNP was precisely described by Homyok et al. (2025). All experimental diets were formulated to provide 3,100 kcal/kg ME and 19% CP for the finisher (d 49 to 70).

At 70 d of age, Iliotibialis muscle samples were collected from 10 females per group. Samples for fatty acid analysis were stored at −20°C. The remaining portions of each sample were immediately frozen in liquid nitrogen and stored at −80°C for subsequent proteomic analysis. Lipids were extracted from approximately 5 g of tissue using chloroform:methanol (2:1, v/v) according to Folch et al. (1957) and methylated following Metcalfe et al. (1966) to generate fatty acid methyl esters (FAMEs). The n-6/n-3 ratio was calculated from chromatogram peak areas. Based on the n-6/n-3 ratio, six samples per group were selected from the initial pool of 10 females to represent the most biologically representative replicates for downstream multivariate analyses and in-depth proteomic profiling (Supplementary Table S1). Proteomic data was subjected to standard quality control procedures, followed by principal component analysis **(PCA)** of global protein profiles. One NC sample was identified as an outlier and was removed from downstream analyses. The same exclusion was applied to the PCA of fatty acid profiles to maintain consistency across datasets. Consequently, the final sample sizes used for fatty acid PCA, and proteomic analysis were *n* = 6 for PC, *n* = 6 for TNP, and *n* = 5 for NC.

### Samples preparation for proteomic analysis

Approximatively 100 mg of each sample were ground and homogenized in liquid nitrogen. Muscle samples were resuspended in 40 mM Tris-HCl (pH 8) (Laville et al., 2009), 2 mM EDTA, and a protease inhibitor cocktail (P2714, Sigma) at the ratio of 1:4 (wt/vol) under agitation (1,000 rpm on ThermoMixer™ C at 4°C), during 30 min. They were then homogenized with a sonicator (5 × 20 s). After homogenization, samples were centrifuged at 10,000 *g* for 10 min at 4°C, and supernatants were stored at −20°C.

Protein concentration was determined using the Uptima BC Assay Protein Quantitation kit (Interchim) according to the manufacturer’s instructions and using bovine serum albumin as the standard.

### Protein inclusion in polyacrylamide gels

Each sample (25 µg) was mixed with 2X sample buffer (125 mM Tris-HCl pH 6.8, 4% SDS, 20% glycerol, 10% TCEP, and 0.004% bromophenol blue), heated at 95°C for 5 min, and loaded on 10% polyacrylamide gels at 50 V for 25 min. The gels were stained overnight with 5% Coomassie Blue R-350 in 30% ethanol/10% acetic acid, followed by destaining. A single protein band per lane was excised for in-gel digestion.

### In-gel digestion

Gel pieces were washed in water:acetonitrile solution (1:1, 5 min) followed by 100% acetonitrile (10 min). Reduction and cysteine alkylation was performed by successive incubation with 10 mM dithiothreitol in 50 mM NH_4_HCO_3_ (30 min, 56°C), then 55 mM iodoacetamide in 50 mM NH_4_HCO_3_ (20 min, RT, in dark). Pieces were then incubated with 50 mM NH_4_HCO_3_ and acetonitrile (1:1, 10 min) followed by acetonitrile (15 min). Proteolytic digestion was carried out overnight using 25 mM NH_4_HCO_3_ with 12.5 ng/μL trypsin (Sequencing grade, Roche diagnostics, Paris, France). Resultant peptides were extracted by incubation in 5% formic acid (sonicated) with the supernatant removed and saved, followed by incubation in water:acetonitrile and 1% formic acid (1:1, 10 min) and a final incubation with acetonitrile (5 min). Again, supernatant was removed and saved. These two peptide extractions were pooled and dried using a SPD1010 speedvac system (Thermosavant, Thermofisher Scientific, Bremen, Germany). The resultant peptide mixture was enriched with SPIN Columns C18 (Millipore) and dried again before being analyzed by nanoflow liquid chromatography tandem mass spectrometry **(nanoLC-MS/MS)**.

### Mass spectrometry LC1D-nanoESI-LTQ-Orbitrap analysis

Peptide mixtures were analyzed by on-line nanoLC-MS/MS. All experiments were performed on a dual linear ion trap Fourier transform mass spectrometer (FT-MS) LTQ Orbitrap Velos Pro (Thermo Fisher Scientific, Bremen, Germany) coupled to an Ultimate® 3000 RSLC Ultra High-Pressure Liquid Chromatographer (Thermo Fisher Scientific, Bremen, Germany) controlled by Chromeleon Software (version 6.80 SR13). Samples were desalted and concentrated for 10 min at 5 µL/min on an LCPackings trap column (Acclaim PepMap 100 C18, 75 µm inner diameter x 2 cm long, 3 µm particles, 100 Å pores). The peptide separation was conducted using a LCPackings nano-column (Acclaim PepMap C18, 75 µm inner diameter x 50 cm long, 2 µm particles, 100 Å pores) at 300 nL/min by applying gradient consisted of 2-45% B during 120 min. Mobile phases consisted of (A) 0.1% formic acid, 97.9% water, 2% acetonitrile (v/v/v) and (B) 0.1% formic acid, 19.9% water, 80% acetonitrile (v/v/v).

Data were acquired using Xcalibur version 3.0.63 software (Thermo Fisher Scientific, San Jose, CA), in positive data-dependent mode in the 300-1,800 m/z mass range. Resolution in the Orbitrap was set at *R* = 60,000. The 20 most intense peptide ions with charge states ≥ 2 were sequentially isolated (isolation width 2m/z, 1 microscan) and fragmented in the high-pressure linear ion trap using CID (collision induced dissociation) mode (collision energy 35%, activation time 10 ms, Qz 0.25). Dynamic exclusion was activated during 30 s with a repeat count of 1. The lock mass was enabled for accurate mass measurements. Polydimethylcyclosiloxane (m/z, 445.120025, (Si(CH_3_)_2_O)_6_) ion was used for internal recalibration of the mass spectra MS/MS ion searches were performed using Mascot search engine version 2.7.0.1 (Matrix Science, London, UK) via Proteome Discoverer 2.5 software (ThermoFisher Scientific, Bremen, Germany) against NCBIprot_Gallus database (2023/01). The search parameters included trypsin as a protease with two allowed missed cleavages and carbamidomethylcysteine, methionine oxidation and acetylation of N-term protein as variable modifications. The tolerance of the ions was set to 5 ppm for parent and 0.8 Da for-fragment ion matches. Mascot results obtained from the target and decoy databases searches were subjected to Scaffold *Q* + *S* v5.2.2 and Scaffold Quant v5.0.3 softwares (Proteome Software, Portland, USA) using the protein cluster analysis option (assemblage of proteins into clusters based on shared peptide evidence). Peptide and protein identifications were accepted if they could be established at greater than 95% probability as specified by the Peptide Prophet algorithm and by the Protein Prophet algorithm, respectively. Protein identifications were accepted if they contained at least two identified peptides.

After applying a protein identification filter to at least 2 unique peptides and 95% probability of identification for peptides and proteins, the data was normalized and quantified according to the "XIC" (eXtracted Ion Chromatogram) method in Scaffold Quant. The "XIC" or "Precursor Intensity (normalized)" is based on the normalized values of the intensities of the chromatographic peaks corresponding to the peptides of a protein. XIC data were Log transformed (Log10 Precursor Intensity), and statistical tests such as Anova and t-test were conducted. A Log2 Fold Change Precursor Intensity was also calculated between 2 conditions to identify the level of abundance of a protein. Proteins were considered differentially expressed if they met the following criteria: a P-value ≤ 0.05 for statistical significance, and a fold change (FC) threshold of < 0.85 (downregulated) or > 1.2 (upregulated). The Log2-transformed fold change (Log2(FC)) corresponding to these thresholds was ≤ −0.234 or ≥ 0.263.

### Principal component analysis of fatty acid profiles

PCA was conducted to evaluate the variation in fatty acid profiles among the experimental groups. The dataset, comprising fatty acid variables and samples, was imported into OriginPro 9.85 (OriginLab, Northampton, Massachusetts, USA) for multivariate analysis.

### Bioinformatics analysis of differential proteins

The thresholds of Log2(FC) ≤ −0.234 or ≥ 0.263 and P-value < 0.05 were set to identify differentially expressed proteins (**DEPs**). Gene ontology (**GO**) enrichment including biological process (**BP**), cellular component (**CC**), molecular function (**MF**) and Kyoto Encyclopedia of Gene and Genomes (**KEGG**) pathways enrichment analyses were performed using ClueGo plug-in via Cytoscape software (version 3.10.3). The GO enriched proteins and KEGG pathways were considered enriched with Right-sided hypergeometric test, a P-value < 0.05, corrected by FDR with Benjamini-Hochberg method (FDR < 0.05). Go terms and KEGG pathways grouping were based on the following criteria: Kappa score threshold was set at 0.45 for functional relatedness, and %Gene/Term was used for term overview. Data were then visualized by ggplot2 package in R version 4.4.2 (R Core Team, 2024). Protein-protein interaction (**PPI**) and MCL clustering was performed via STRING (version 12.0, https://string-db.org) against the *Gallus gallus* database (2023/01) and considering a high confidence score of 0.4 for interaction.

## Results

### Principal component analysis (PCA) of fatty acid profiles in Korat chicken received tuna oil in lipid nanoparticles

**Fig. 1:**
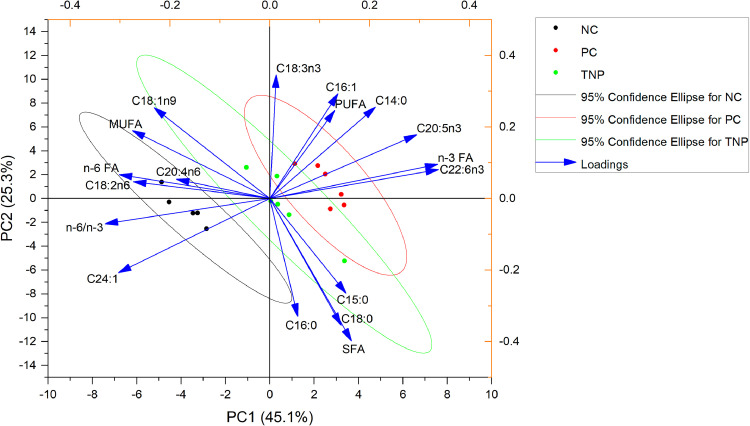
Principal Component Analysis (PCA) of fatty acid profiles from experimental groups. The biplot represents the distribution of fatty acids across three dietary treatments: Negative control (NC, *n* = 5), Positive control (PC, 3% tuna oil, *n* = 6), and Targeted-lipid nanoparticles (TNP, 3% tuna oil in lipid nanoparticles, *n* = 6). Each point represents a sample, with NC indicated by black circles, PC by red circles, and TNP by green circles. The ellipses denote 95% confidence intervals for each group. The vectors (blue arrows) correspond to specific fatty acids and illustrate their contribution to the first two principal components, PC1 (45.1% of total variance) and PC2 (25.3% of total variance).

The PCA biplot of fatty acid profiles (Fig. 1) exhibited the effects of dietary n-3 PUFA supplementation on Korat chicken thigh meat. Distinct separations among groups accounted for 70.4% of the total variance. PC1 (45.1%) predominantly reflected variations in PUFAs, particularly for n-3 fatty acids EPA (C20:5n3) and DHA (C22:6n3), as well as n-6 fatty acids linoleic acid (C18:2n6) and arachidonic acid (C20:4n6). PC1 differentiated the NC group from the n-3 PUFA-supplemented groups and further separated the TNP from the PC group, suggesting that nanoencapsulation enhanced the bioavailability and tissue incorporation of n-3 PUFAs. PC2 (25.3%) highlighted differences in saturated fatty acids (C14:0, C16:0, C18:0) and monounsaturated fatty acids (C16:1, C18:1n9), distinguishing the NC group from the supplemented groups.

### Differential protein expression in thigh muscle

#### Principal component analysis

**Fig. 2:**
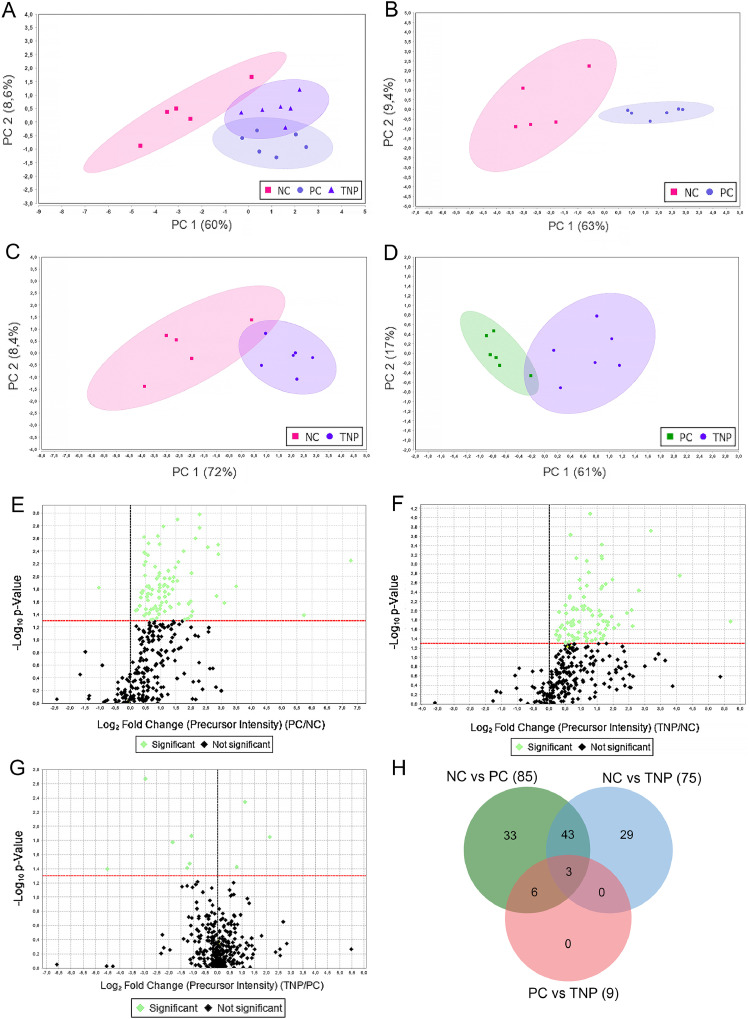
Principal Component Analysis (PCA) plots (A, B, C and D), illustrating the separation of dietary treatment groups based on differential protein expression profiles including NC (6% rice bran oil), PC (3% tuna oil), and TNP (3% tuna oil loaded in lipid nanoparticles). Volcano plots (E, F, and G) and Venn diagram (H) illustrating of the three pairwise groups. The ellipses in PCA plot represent the 95% confidence intervals for each group, indicating the variability and clustering within each treatment based on differentially expressed proteins. For the volcano plot, the x-axis represents the log2(fold change) in protein abundance, and the y-axis represents the -log10(p-value). Proteins with significant differential expression are highlighted in green, while non-significant proteins are shown in black. The red horizontal line indicates the significance threshold for p-values.

PCA was performed to evaluate the effects of dietary n-3 PUFA supplementation on protein expression in thigh meat of Korat chickens. Differentially expressed proteins are presented in the Supplementary Table S2. In the overall PCA including all treatments (NC, PC, and TNP; Fig. 2A), clear separation among the three groups was observed. PC1 explained 60% of the total variance and effectively separated the PC group from NC and TNP, whereas PC2 accounted for 29% of the variance and differentiated TNP from NC.

Pairwise PCA comparisons further demonstrated treatment-dependent differences in protein expression. In the NC vs. PC comparison (Fig. 2B), samples were distinctly separated along PC1, which explained 63% of the variance, indicating substantial proteomic changes associated with free tuna oil supplementation. The comparison between NC and TNP (Fig. 2C) showed a marked separation, which PC1 accounted for 72% of the variance, reflecting the strong effect of nanoencapsulated n-3 PUFAs relative to the control diet. Despite identical n-3 PUFA content, the PCA comparing PC and TNP groups (Fig. 2D) revealed distinct clustering between treatments, with PC1 explaining 61% of the variance, indicating that nanoencapsulation modified protein expression profiles compared with free tuna oil.

The 95% confidence ellipses indicated low within-group variability and clear separation among treatments, supporting the consistency and reproducibility of the proteomic responses to each dietary intervention.

### Volcano plots, heatmap and Venn diagram

**Fig. 3:**
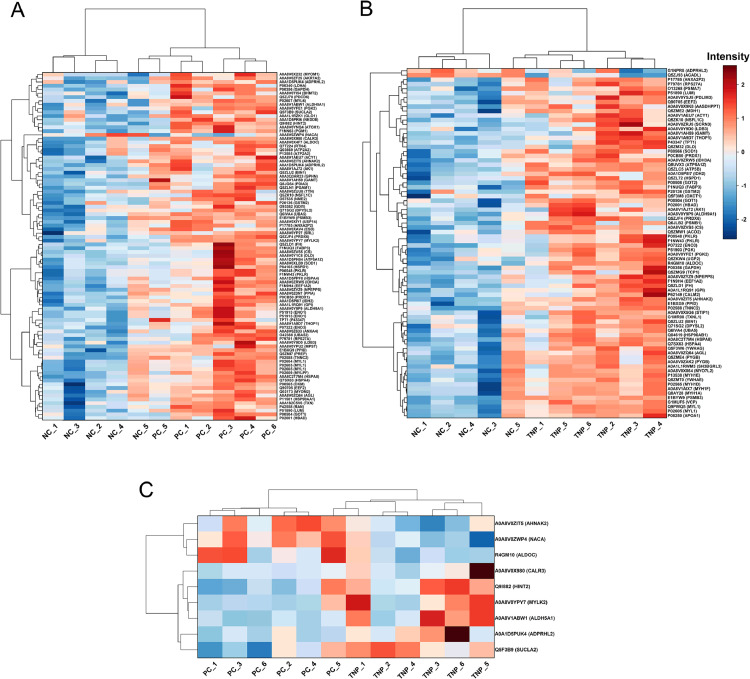
Heatmap representations of differentially expressed proteins across sample groups: (A) Negative control vs. Positive control (NC vs. PC); (B) Negative control vs. targeted lipid nanoparticles (NC vs. TNP); and (C) Positive control vs. targeted lipid nanoparticles (PC vs. TNP). Samples are displayed along the x-axis, while proteins are listed on the y-axis. Hierarchical clustering is illustrated by dendrograms on both axes, grouping proteins and samples according to similarities in expression patterns. The color scale adjacent to each heatmap denotes relative protein expression levels, ranging from low (blue) to high (red).

Volcano plots (Fig.s 2E, 2F, and 2G) and a heatmap (Fig. 3) were used to illustrate differential protein expression among dietary treatment groups based on pairwise comparisons (NC vs. PC, NC vs. TNP, and PC vs. TNP). The volcano plots identified numerous differentially abundant proteins (**DAPs**), with 92 proteins detected in the PC vs. NC comparison, 78 proteins in NC vs. TNP comparison, and 9 proteins in the PC vs. TNP comparison. Statistically significant proteins are highlighted in green.

In the volcano plots, the x-axis represents the fold change (**FC**) in protein abundance, while the y-axis represents the -log10 (p-value). Several proteins associated with n-3 PUFA supplementation exhibited significant upregulation, with FC values greater than 1.2, and many proteins exceeded the statistical significance threshold indicated by the red horizontal line.

The heatmap (Fig. 3) further illustrated distinct expression patterns across dietary treatments, showing clear clustering of samples according to their protein expression profiles. Notably, the n-3 PUFA-supplemented groups (PC and TNP) were clearly separated from the NC, indicating consistent alterations in protein expression induced by dietary supplementation.

The Venn diagram (Fig. 2H; protein lists are provided in Supplementary Table S2) showed the distribution and overlap of DAPs identified from pairwise dietary treatment comparisons. A total of 85 and 75 DAPs were detected in the NC vs. PC and NC vs. TNP comparisons, respectively, with 43 proteins shared between the two comparisons, indicating common changes in protein expression in response to both free and encapsulated tuna oil supplementation. In contrast, only 9 proteins were differentially abundant between the PC and TNP groups. Additionally, 33 and 29 proteins were uniquely identified in the NC vs. PC and NC vs. TNP comparisons, respectively, suggesting treatment-specific effects on protein expression. To capture the overall proteomic response to dietary supplementation, all DAPs identified from the pairwise comparisons (NC vs. PC, NC vs. TNP, and PC vs. TNP) were included in subsequent analyses.

### Biological responses to dietary interventions

To characterize the biological responses to dietary treatments, all DEPs identified from the pairwise comparisons (NC vs. PC, NC vs. TNP, and PC vs. TNP) were subjected to GO and KEGG pathway enrichment analyses. For the NC vs. PC comparison, enrichment analysis identified 152 BP, 16 CC, 24 MF, and 21 KEGG pathways. Similarly, the NC vs. TNP comparison yielded 167 BP, 15 CC, 22 MF, and 21 KEGG pathways (Supplementary Table S3).

Enriched GO terms and KEGG pathways were observed in both the NC vs. PC and NC vs. TNP comparisons, whereas no significant enrichment was detected in the PC vs. TNP comparison. This finding indicates that, although minor differences in protein abundance were observed between the PC and TNP groups, these differences were not sufficient to result in enriched biological categories.

To further understand the molecular responses from dietary interventions, we used ClueGO plug-in to group enriched GO terms and KEGG pathways from the NC vs. PC and NC vs. TNP comparisons. This analysis categorizes terms into functional groups, highlighting differences in metabolic, cellular, and molecular processes.

### Functional groups of biological responses to dietary treatments: Insights from GO and KEGG pathway clustering

**Fig. 4:**
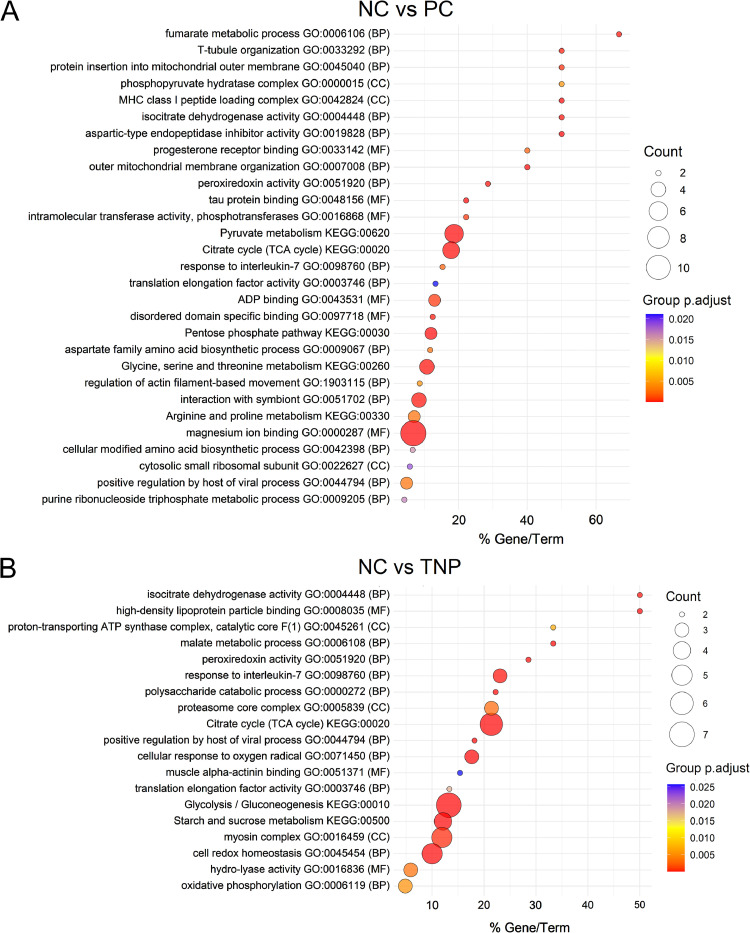
Comparative analysis of GO enrichment terms and KEGG pathways involvement in dietary treatments, NC vs. PC (A) and NC vs. TNP (B).

Clustering analysis of enriched GO terms and KEGG pathways highlighted distinct functional groups associated with each dietary treatment. The grouped biological responses are summarized in Fig. 4, with detailed enrichment information provided in Supplementary Table S4.

For the NC vs. PC comparison, enriched GO terms and KEGG pathways were classified into seven functional categories based on group-adjusted p-values using the ClueGO plugin. These categories included: (1) central metabolism and energy production; (2) amino acid metabolism and protein biosynthesis and inhibition; (3) cellular structure and dynamics; (4) binding and transport mechanisms; (5) stress response and immune function; (6) nucleotide metabolism and ribosomal activity; and (7) specialized cellular functions and interactions.

Similarly, the NC vs. TNP comparison yielded seven categories. Enriched terms and pathways were grouped into: (1) energy production and mitochondrial function; (2) carbohydrate and energy metabolism; (3) protein and amino acid metabolism; (4) cellular stress response and redox homeostasis; (5) muscle function and structural integrity; (6) lipid transport and metabolic regulation; and (7) immune response.

Comparison of pathway enrichment between the two n-3 PUFA supplementation strategies revealed both shared and distinct functional groupings, indicating differential engagement of biological processes depending on the form of n-3 PUFA delivery.

### Interaction network of DEPs related to biological function in each comparison

**Fig. 5:**
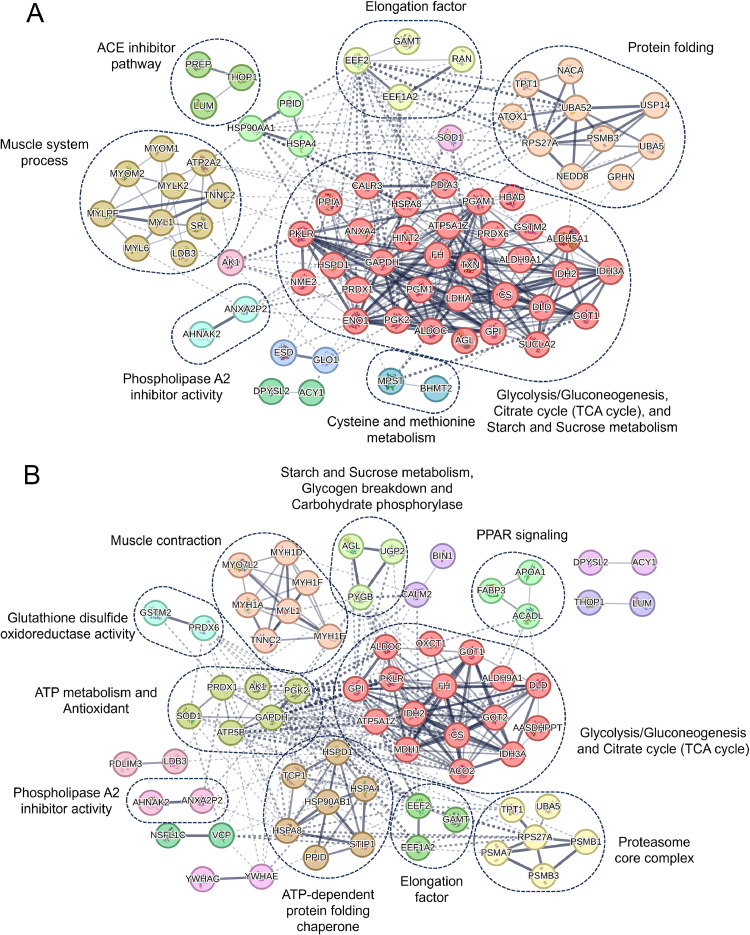
Protein-protein interaction in pairwise dietary treatments, NC vs. PC (A) and NC vs. TNP (B).

Protein-protein interactions (PPI) network analysis revealed that most DEPs interacted with one another and formed distinct functional clusters in each comparison (Fig. 5; Supplementary Table S5).

In the NC vs. PC comparison, major interaction clusters were related to glycolysis, TCA cycle, and starch/sucrose metabolism. A comparable metabolic cluster was also observed in the NC vs. TNP comparison, with enrichment of glycolysis-related enzymes. However, proteins involved in starch/sucrose metabolism were limited to glycogen breakdown and carbohydrate phosphorylase, with only UGP2 and PYGB identified. Additionally, the NC vs. TNP comparison revealed interaction clusters related to lipid metabolism for energy production, including proteins involved in high-density lipoprotein particle binding and PPAR signaling.

Clusters associated with oxidative stress and antioxidant activity were identified in both dietary comparisons. In the NC vs. PC comparison, antioxidant-related proteins formed distinct clusters, whereas in the NC vs. TNP comparison, antioxidant enzymes were grouped together with ATP metabolism-related proteins, suggesting coordinated involvement in redox balance and energy production. Both n-3 PUFA dietary treatments were associated with enhanced antioxidant-related protein interactions, with glutathione-related pathways uniquely observed in the TNP group.

Proteins related to muscle function were also identified in the interaction networks. In the NC vs. PC comparison, muscle-related clusters were primarily associated with structural regulation. In contrast, the NC vs. TNP comparison displayed a prominent muscle-related cluster including MYH1, MYO7L2, MYL1, and TNNC2, indicating enrichment of proteins involved in muscle contraction and structural integrity.

Immune-related interaction clusters were evident in the NC vs. PC comparison, with proteins associated with antigen processing and cytokine response pathways. Notably, HSP90AA1, CALR3 and PDIA3 were involved in antigen presentation through the MHC class I peptide-loading complex (PLC). In the NC vs. TNP comparison, immune-related proteins such as HSPA8 and STIP1 were also identified; however, these formed smaller interaction clusters.

Finally, proteins associated with protein folding and translation elongation were observed in the NC vs. PC comparison. In the NC vs. TNP comparison, proteasome proteins and chaperones formed distinct clusters. These results indicated that both dietary n-3 PUFA treatments influenced protein homeostasis, with proteasome-related protein interactions being more prominent in the TNP group.

## Discussion

### Lipid nanoparticle-delivered n-3 PUFAs are associated with a shift toward mitochondria-centered metabolic remodeling versus free oil

The integrated fatty acid and proteomic profiles supported the concept that replacing half of the dietary n-6 PUFAs with n-3 PUFAs reshaped metabolic pathways in skeletal muscle. Fatty acid PCA clearly separated both PC and TNP groups from the NC group, reflecting increased n-3 PUFAs and a reduced n-6/n-3 ratio (Fig. 1). Consistent with this shift in lipid composition, the majority of DAPs were identified in the NC vs. PC and NC vs. TNP comparisons (Fig. 2E and 2F). Functional enrichment analyses, including GO, KEGG, and PPI network analyses, revealed that these n-3 PUFA-responsive proteins were predominantly associated with energy metabolism-related pathways, such as glycolysis/gluconeogenesis, the TCA cycle, oxidative phosphorylation, and amino acid metabolism (Fig.s 4 and 5; Supplementary Table S5).

Within this shared metabolic response to n-3 PUFA enrichment, clear distinctions emerged between lipid delivery forms. Tuna oil encapsulated in lipid nanoparticles (TNP) was associated with a proteomic signature indicative of enhanced mitochondria-centered aerobic metabolism, characterized by greater reliance on oxidative pathways. In contrast, free tuna oil (PC) retained a metabolic profile more consistent with glycolytic activity and rapid energy-buffering systems. These findings suggest that lipid nanoparticle-mediated delivery of n-3 PUFAs may promote more efficient mitochondrial metabolic remodeling in skeletal muscle compared with free oil supplementation.

In the PC group, the abundance patterns of amino acid- and energy-related proteins were consistent with a predominantly glycolytic and rapid-buffering configuration. Creatine kinase M-type (**CKM**) and guanidinoacetate N-methyltransferase (**GAMT**) support the creatine-phosphocreatine system, which enables rapid ATP regeneration under conditions of high or fluctuating energy demand (Schulze et al., 2016; Gerling et al., 2019; Tomczyk, 2024). In parallel, betaine-homocysteine S-methyltransferase 2 (**BHMT2**) catalyzes the remethylation of homocysteine to methionine, which is subsequently converted to S-adenosylmethionine **(SAM)**, a universal methyl donor required for creatine synthesis and other methylation-dependent pathways (Nabuurs et al., 2013; Garibotto et al., 2023). Cytosolic aspartate aminotransferase (**GOT1**) links amino acid metabolism to the TCA cycle by channeling aspartate into oxaloacetate (Li and Hoppe, 2023), whereas phosphoglycerate mutase 1 (**PGAM1**) and dihydrolipoyl dehydrogenase (**DLD**) support glycolytic throughput and oxidative metabolism, thereby supplying ATP and metabolic intermediates for amino acid biosynthesis and other anabolic processes (Hitosugi et al., 2012; Yang et al., 2019; Katsnelson and Ceddia, 2020; Li and Liu, 2020).

In addition, the upregulation of glycogen debranching enzyme (**AGL**) and fructose-bisphosphate aldolase C (**ALDOC**) in the PC vs. NC comparison indicates enhanced glycogen mobilization and glycolytic entry, providing glucose-derived substrates to sustain this rapid-buffering metabolic configuration. This pattern is compatible with previous proteomic evidence indicating that n-3 PUFAs can modulate carbohydrate and energy metabolism (Ahmed et al., 2014). Notably, lactoylglutathione lyase (**GLO1**) was upregulated in the PC group but was not differentially abundant in the NC vs. TNP comparison. This observation raises the possibility of increased engagement of the glyoxalase system to detoxify methylglyoxal **(MGO)**, a reactive dicarbonyl generated largely from glycolytic triose phosphates, thereby limiting downstream glycation and carbonyl stress (Allaman et al., 2015; Syed et al., 2023). Nevertheless, targeted quantification of MGO and glycation adducts will be required to validate this inference. Collectively, these features suggest that after 21 d of feeding, skeletal muscles in the PC group adapted toward a flexible glycolysis-creatine buffering strategy to sustain ATP supply.

By contrast, the TNP group showed a proteomic signature consistent with enhanced mitochondrial efficiency. Notably, l-lactate dehydrogenase A chain (**LDHA**) and succinate-CoA ligase (**SUCLA2**) were not identified as differentially abundant in the TNP vs. NC comparison (Supplementary Table S5), suggesting a reduced dependence on anaerobic/substrate-level ATP synthesis (Wang et al., 2017; Jannas-Vela et al., 2023). In parallel, higher abundance of key oxidative enzymes, including isocitrate dehydrogenase subunit alpha (**IDH3A**), DLD, and fumarate hydratase (**FH**) was consistent with increased TCA-cycle flux and efficient NAD⁺/NADH turnover (Lalia et al., 2017).

Moreover, higher levels of ATP synthase subunit beta (**ATP5B**), aconitase 2 (**ACO2**), malate dehydrogenase 1 (**MDH1**), and aspartate aminotransferases GOT1 and GOT2, together with PPAR-associated proteins such as apolipoprotein A-I (**APOA1**) and long-chain acyl-CoA dehydrogenase (**ACADL**), further supported this metabolic shift by strengthening mitochondrial-cytosolic coupling, including the malate-aspartate shuttle, and enhancing fatty acid β-oxidation. These coordinated changes are indicative of optimized aerobic energy flux and mitochondrial integration (Ahmed et al., 2014; Tachtsis et al., 2018; Broeks et al., 2021; Jannas-Vela et al., 2023).

Consistent with this oxidative phenotype, the upregulation of glycogen phosphorylase (**PYGB**), AGL, UTP-glucose-1-phosphate uridylyltransferase (**UGP2**), and ALDOC in the TNP vs. NC comparison indicates active glycogen mobilization and glycolytic entry, providing aerobic substrates to support mitochondrial ATP production (Holness and Sugden, 2003; Hargreaves and Spriet, 2020). Finally, the relatively lower prominence of CKM in the TNP group, together with enrichment of oxidative and lipid-linked proteins, suggests reduced reliance on the creatine-phosphocreatine buffering system and a metabolic preference for sustained ATP generation via oxidative phosphorylation coupled to fatty acid β-oxidation (Tomczyk, 2024).

These proteomic features are compatible with delivery format-dependent differences in the incorporation and utilization of n-3 PUFAs as metabolic substrates in skeletal muscle. Although the PC group showed higher absolute n-3 PUFA deposition (Figure 1; Supplementary Table S1), its proteomic profile remained predominantly glycolytic and appeared to rely more heavily on rapid ATP regeneration through glycolytic pathways and the creatine-phosphocreatine system. In contrast, muscle from TNP-fed chickens exhibited a metabolic phenotype indicative of enhanced mitochondrial oxidative capacity and improved aerobic efficiency after 21 d of feeding. These distinctions likely reflect steady-state metabolic adaptations to the respective lipid delivery formats rather than acute post-prandial responses.

Moreover, the greater reliance on oxidative phosphorylation observed in the TNP group is consistent with our previous findings of increased hemoglobin and hematocrit following lipid nanoparticle supplementation, which are compatible with enhanced oxygen transport capacity (Homyok et al., 2025). Nevertheless, the causal links between encapsulated n-3 PUFA delivery, mitochondrial aerobic remodeling, and oxygen utilization remain hypothetical and will require targeted functional validation in future studies.

### Lipid nanoparticles attenuate antioxidant and detoxification responses compared with free tuna oil

Relative to the n-6 PUFA-rich control, both forms of n-3 PUFA supplementation elicited an antioxidant and detoxification response in skeletal muscle (Figure 5; Supplementary Table S5). However, within this shared n-3 PUFA-responsive context, the free-oil PC treatment was associated with a broader and more robust redox-maintenance signature, whereas lipid nanoparticle delivery (TNP) induced a more restrained redox adjustment. This distinction is consistent with the greater susceptibility of unencapsulated PUFAs to lipid peroxidation and the concurrent shift toward mitochondria-centered oxidative metabolism under TNP supplementation.

The PC group showed a stronger induction of both first-line ROS/peroxide-scavenging enzymes and supporting redox-recycling/detoxification components. Superoxide dismutase (**SOD1**) provides early defense against superoxide radicals (O₂⁻•) (Kusunoki et al., 2013), while peroxiredoxin-1 (**PRDX1**) and peroxiredoxin-6 (**PRDX6**) reduce hydrogen peroxide (H₂O₂) and lipid hydroperoxides (ROOH), thereby limiting damage to cellular macromolecules (Burillo et al., 2016; Jackson et al., 2022). This peroxiredoxin system is supported by redox-recycling components, with thioredoxin (**TXN**) sustaining peroxiredoxin turnover via disulfide bond reduction (AlOkda and Van Raamsdonk, 2023), and S-formylglutathione hydrolase (**ESD**) contributing to downstream detoxification of aldehyde-related intermediates (Gonzalez et al., 2006). Furthermore, glutathione S-transferase 2-like (**GSTM2**) is consistent with detoxification of reactive lipid peroxidation-derived aldehydes (e.g., 4-HHE/4-HNE) via glutathione conjugation (Balogh and Atkins, 2011; Singhal et al., 2015), while ADP-ribosylarginine hydrolase (**ADPRHL2**) may contribute to the repair of stress-modified proteins by reversing ADP-ribose adducts (Beijer et al., 2021).

Moreover, the PC group also showed enrichment of enzymes that can operate sequentially to detoxify lipid-derived aldehydes. Aflatoxin B1 aldehyde reductase member 2 (**AKR7A2**) can reduce reactive aldehydes (e.g., 4-HHE) to less reactive alcohols (Barski et al., 2008; Penning, 2015; Mol et al., 2017). Aldehyde dehydrogenase, mitochondrial (**ALDH2**), together with 4-trimethylaminobutyraldehyde dehydrogenase (**ALDH9A1**), then oxidize aldehydes and alcohol intermediates, facilitating safer clearance (Vistoli et al., 2013; Ahmed Laskar and Younus, 2019; Karan et al., 2023). In the present study, these protein expression patterns are consistent with activation of an aldehyde-detoxification cascade in the PC group. However, direct profiling of lipid peroxidation-derived aldehydes and assessment of their correlations with the abundance of these enzymes will be required to substantiate this mechanism.

By contrast, the TNP group exhibited a more streamlined redox profile under encapsulated delivery. SOD1 remained central for superoxide neutralization, while PRDX1 and PRDX6 were differentially abundant in this comparison pairwise but with more modest changes than observed under free-oil delivery. TXN was not differentially abundant, whereas thioredoxin-like protein 1 (**TXNL1**) was upregulated, which may help regulate the thioredoxin-peroxiredoxin axis (Zhao and Qi, 2021; Andor et al., 2023). SH3 domain-binding glutamic acid-rich protein (**SH3BGRL3**), a thioredoxin-like adaptor implicated in redox-sensitive signaling (Liu et al., 2016), was also upregulated and may further modulate this redox regulation under nanoparticle delivery. On the aldehyde-detoxification axis, ALDH9A1 and GSTM2 were upregulated, whereas AKR7A2 and ALDH2 were not differentially abundant, AKR7A2 and ALDH2 were not differentially abundant, indicating a less extensive aldehyde-detoxification response than under free-oil delivery. However, the proposed links between these protein expression patterns and specific oxidative species under differential n-3 PUFA delivery remain inferential and will require targeted quantification.

### Delivery format links redox burden to membrane phospholipid maintenance and lipid trafficking

Building on the redox/detoxification differences described above, oxidative pressure in n-3 PUFA-fed muscle would be expected to impact the muscle membrane, where lipid peroxidation can generate oxidized phospholipids that compromise membrane integrity and necessitate coordinated repair and lipid-handling responses.

In the PC group, annexin A2 (**ANXA2P2**), annexin A4 (**ANXA4**), protein AHNAK2 (**AHNAK2**), programmed cell death 6-interacting protein (**PDCD6)**, and peroxiredoxin 6 (**PRDX6**) were differentially abundant, supporting a Ca²⁺-linked membrane “damage-control” and phospholipid-maintenance module. Annexins are oxidant- and Ca²⁺-responsive membrane-binding proteins: ANXA2P2 can stabilize injured membrane regions and bind peroxidized phospholipids, whereas ANXA4 may further reinforce membrane stabilization during Ca²⁺ flux. Together, these actions may limit the spread of lipid peroxidation and provide a platform for repair (Monastyrskaya et al., 2009; Jaiswal et al., 2014; Hajjar et al., 2015; Demonbreun et al., 2016; Defour et al., 2017; Dallacasagrande and Hajjar, 2020). AHNAK2, a cytoskeletal organizer, is compatible with strengthened membrane-cytoskeletal coupling and vesicle trafficking capacity that supports membrane remodeling and phospholipid redistribution (Jaiswal et al., 2014; Zhang et al., 2023; Prislusky et al., 2024). PDCD6 is a Ca²⁺-binding regulator linked to trafficking programs that can contribute to membrane turnover to repair (Zhen et al., 2021; Drescher et al., 2023). Notably, PRDX6 provides a mechanistic bridge between redox stress and membrane phospholipid maintenance through its dual PLA2 and glutathione peroxidase-like activities, which can facilitate removal/repair of oxidized phospholipids (Fisher, 2017; Manson et al., 2023). Overall, these changes are compatible with stronger engagement of membrane stabilization and phospholipid quality-control processes under free-oil delivery, aligning with the broader antioxidant/detoxification signature observed in PC.

In the TNP condition, ANXA2P2 and PRDX6 were differentially abundant, implicating membrane-associated phospholipid maintenance under n-3 PUFA supplementation. However, ANXA4 and PDCD6 were not differentially abundant in this contrast, suggesting that the TNP-associated response is less characterized by Ca²⁺-triggered lesion-patching programs and more consistent with membrane stabilization and lipid-cargo trafficking. The marked increase in AHNAK2 is particularly notable and is compatible with cytoskeletal reorganization and vesicle trafficking demands that can accompany nanoparticle-membrane interactions and intracellular routing of lipid payloads (Jaiswal et al., 2014; Zhang et al., 2023; Prislusky et al., 2024). Although proteins directly mediating nanoparticle uptake were not identified in this dataset, prior work indicates that endocytic processes contribute to nanoparticle internalization and can be influenced by particle properties and protein corona formation, potentially shaping downstream trafficking routes (Yeh et al., 2014; Foroozandeh and Aziz, 2018; Wang et al., 2021; Qin et al., 2020; Baker et al., 2022; Wang et al., 2024). With this trafficking-oriented interpretation, chaperonin-containing TCP1 **(CCT)** components such as T-complex protein 1 subunit alpha (**TCP1**) can support folding/maintenance of cytoskeletal proteins required for vesicle transport (Dmitrieff and Nédélec, 2016; Mulens-Arias, 2021; Cui et al., 2022). Moreover, APOA1 was differentially abundant in the NC vs. TNP comparison and may contribute to lipid redistribution and phospholipid handling through lipid-transport pathways (Caracciolo, 2015; Chen et al., 2022). Fatty acid-binding protein 3 (**FABP3**) supports intracellular fatty-acid binding/trafficking and may channel n-3 PUFAs toward membrane remodeling or β-oxidation depending on cellular context (Huang et al., 2018; Lee et al., 2020). The membrane-associated signatures support a delivery-format effect in which nanoparticle delivery favors organized lipid handling/trafficking over a broad Ca²⁺-linked membrane-repair. In contrast, the free-oil profile is more compatible with higher phospholipid stress and stronger engagement of Ca²⁺-linked stabilization/repair and oxidized-phospholipid handling. Nevertheless, these interpretations remain inferential and would benefit from targeted validation (e.g., oxidized-phospholipid profiling, membrane integrity assays, Ca²⁺ flux measurements, and uptake/trafficking perturbation experiments).

### Unencapsulated versus nanoparticle n-3 PUFA delivery differentially couples Ca²⁺ handling to distinct proteostasis programs

In the NC vs. PC comparison, the DAPs set included sarcoplasmic/endoplasmic reticulum calcium ATPase 2 (**ATP2A2**) and sarcalumenin (**SRL**), suggesting increased sarcoplasmic-reticulum Ca²⁺ reuptake and buffering capacity during contraction-relaxation cycling. Sarcomeric reinforcement was further reflected by myomesin-1 (**MYOM1**) and myomesin-2 (**MYOM2**), which stabilize thick filaments at the M-line and support force transmission and recovery (Prill et al., 2019; Hang et al., 2021; Lamber et al., 2022). In addition, myosin light polypeptide 6 (**MYL6**) together with myosin light chain kinase 2 (**MYLK2**) and myosin regulatory light chain 11 (**MYLPF**) points to enhanced Ca²⁺-dependent tuning of actomyosin activation that may help preserve contractile function under stress (Stull et al., 2011; Murgia et al., 2021). Beyond the contractile/Ca²⁺-handling module, calreticulin 3 (**CALR3**) and protein disulfide-isomerase A3 (**PDIA3**) were differentially abundant only in NC vs. PC (Figure 4; Supplementary Table S4), these proteins mapped to the MHC class I peptide loading complex (GO:0042824), consistent with increased engagement of ER luminal Ca²⁺-linked folding and disulfide-bond quality control under stress conditions (Zhang et al., 2019; Antoniotti et al., 2022; Michalak, 2024). The same contrast also contained ubiquitin processing components including ubiquitin-40S ribosomal protein S27a (**RPS27A**), ubiquitin-60S ribosomal protein L40 (**UBA52**), and ubiquitin carboxyl-terminal hydrolase 14 (**USP14**), supporting a working hypothesis of increased ubiquitin-dependent handling of damaged or misfolded proteins, consistent with elevated proteostasis pressure (Colberg et al., 2020). The co-occurrence of Ca²⁺-handling/sarcomeric-stabilization proteins with ER folding and ubiquitin-processing signals supports a model in which unencapsulated n-3 PUFA delivery is associated with stronger lipid-oxidation-linked cellular stress that may secondarily perturb Ca²⁺ homeostasis and drive compensatory remodeling; however, direct evidence for enhanced antigen presentation or immune-surveillance activation will require targeted validation.

By contrast, the NC vs. TNP contrast supports a working hypothesis of a more performance-oriented remodeling under nanoparticle delivery. Here, PDZ and LIM domain protein 3 (**PDLIM3**) was differentially abundant together with fast contractile components including fast myosin heavy chain HCIII (**MYH1A**), myosin-1B (**MYH1D**), myosin heavy chain 1E (**MYH1E**), and myosin heavy chain 1F (**MYH1F**), a pattern compatible with remodeling of the contractile apparatus toward faster, high-power function (Krcmery et al., 2010; Lee et al., 2019; Yin et al., 2020; Jannas-Vela et al., 2023). In the same contrast, stress-induced phosphoprotein 1 (**STIP1**) was differentially abundant, and given that STIP1/Hop coordinates Hsp70-Hsp90 chaperone cycles and can influence client-protein triage, its co-occurrence with the TNP remodeling module is consistent with a hypothesis that nanoparticle delivery places a stronger emphasis on cytosolic chaperone coordination during remodeling and intracellular handling of lipid cargo (Suzuki et al., 2003; Nemoto et al., 2006; Bhattacharya et al., 2020).

Across both pairwise contrasts (NC vs. PC and NC vs. TNP), heat shock protein family A member 4 (**HSPA4**) and heat shock cognate 71 kDa protein (**HSPA8**) was differentially abundant in our dataset, indicating that n-3 PUFA supplementation is accompanied by increased cytosolic chaperone demand relative to the n-6-rich NC background. Notably, the proteostasis context diverged by delivery format. In NC vs. PC, HSPA4/HSPA8 co-occurred with ER/SR-associated folding factors including calreticulin 3 (**CALR3**) and protein disulfide-isomerase A3 (**PDIA3**) together with ubiquitin-related processing components (**RPS27A, UBA52, USP14**). Because calreticulin-family proteins and PDIA3 contribute to ER luminal Ca²⁺-linked folding and disulfide-bond quality control, this PC-specific combination supports a working hypothesis of broader ER/SR-centered protein quality-control engagement under higher cellular stress (Zhang et al., 2019; Antoniotti et al., 2022; Michalak, 2024). In contrast, NC vs. TNP lacked this CALR3/PDIA3-plus-ubiquitin cluster while showing STIP1, supporting a more specific hypothesis that the dominant proteostasis requirement under nanoparticle delivery is cytosolic chaperone-cycle coordination rather than a prominent ER/SR stress escalation. Nonetheless, this interpretation is hypothesis-generating and warrants focused on delivery format validation, for example by comparing ER/SR stress and ubiquitin-proteasome flux markers with assays of STIP1-Hsp70/Hsp90 complex formation (and perturbation), alongside labeled-nanoparticle uptake/trafficking readouts to directly link the TNP format to chaperone-cycle coordination.

## Conclusions

Comparative proteomics revealed both shared and delivery-specific muscle responses to n-3 PUFA supplementation in slow-growing chickens. Across PC and TNP, differentially abundant proteins were enriched for energy-related processes, redox regulation, and stress/immune functions, indicating a common adaptive signature. Notably, the PC condition showed broader enrichment involving amino acid metabolism, MHC-related and proteostasis pathways, and calcium/structural remodeling, whereas the TNP condition displayed a more focused pattern emphasizing mitochondrial oxidative metabolism, lipid transport/β-oxidation, and carbohydrate-associated substrate supply. These findings are based on pathway-level proteomic patterns and should be interpreted as mechanistic and hypothesis-generating. Targeted validation (e.g., mitochondrial function/enzyme assays and orthogonal confirmation of key proteins) is required to establish functional relevance and clarify delivery-specific physiological implications.

## CRediT authorship contribution statement

**Piyaradtana Homyok:** Data curation, Formal analysis, Funding acquisition, Investigation, Methodology, Project administration, Resources, Software, Validation, Visualization, Writing – original draft, Writing – review & editing. **Pramin Kaewsatuan:** Methodology, Supervision, Writing – review & editing. **Valérie Labas:** Data curation, Formal analysis, Investigation, Software, Validation, Writing – review & editing. **Daniel Tomas:** Data curation, Formal analysis, Investigation, Software, Validation, Writing – review & editing. **Ana Paula Teixeira-Gomes:** Investigation, Project administration, Resources, Software, Validation, Writing – review & editing, Data curation, Formal analysis. **Elisabeth Baéza:** Investigation, Methodology, Project administration, Resources, Supervision, Validation, Visualization, Writing – original draft, Writing – review & editing. **Cécile Berri:** Supervision, Validation, Visualization, Writing – original draft, Writing – review & editing. **Amonrat Molee:** Conceptualization, Funding acquisition, Methodology, Supervision. **Wittawat Molee:** Funding acquisition, Methodology, Supervision, Validation, Writing – original draft, Writing – review & editing.

## Disclosures

The authors declare no conflict of interest.

## Appendix Supplementary materials

Image, application 1

Image, application 2

Image, application 3

Image, application 4

Image, application 5

## References

[bib0001] Ahmed Laskar A., Younus H. (2019). Aldehyde toxicity and metabolism: the role of aldehyde dehydrogenases in detoxification, drug resistance and carcinogenesis. Drug Metab. Rev..

[bib0002] Ahmed A.A., Balogun K..A., Bykova N.V., Cheema S.K. (2014). Novel regulatory roles of omega-3 fatty acids in metabolic pathways: a proteomics approach. Nutr. Metab. (Lond)..

[bib0003] Allaman I., Bélanger M., Magistretti P.J. (2015). Methylglyoxal, the dark side of glycolysis. Front. Neurosci..

[bib0004] AlOkda A., Van Raamsdonk J.M. (2023). Evolutionarily conserved role of thioredoxin systems in determining longevity. Antioxidants (Basel).

[bib0005] Andor A., Mohanraj M., Pató Z.A., Úri K., Biri-Kovács B., Cheng Q., Arnér E.S.J. (2023). TXNL1 has dual functions as a redox active thioredoxin-like protein as well as an ATP- and redox-independent chaperone. Redox Biol.

[bib0006] Antoniotti V., Bellone S., Gonçalves Correia F.P., Peri C., Tini S., Ricotti R., Mancioppi V., Gagliardi M., Spadaccini D., Caputo M., Corazzari M., Prodam F. (2022). Calreticulin and PDIA3, two markers of endoplasmic reticulum stress, are associated with metabolic alterations and insulin resistance in pediatric obesity: a pilot study. Front. Endocrinol. (Lausanne)..

[bib0007] Baker A.N., Hawker-Bond G..W., Georgiou P.G., Dedola S., Field R.A., Gibson M.I. (2022). Glycosylated gold nanoparticles in point of care diagnostics: from aggregation to lateral flow. Chem. Soc. Rev..

[bib0008] Balogh L.M., Atkins W.M. (2011). Interactions of glutathione transferases with 4-hydroxynonenal. Drug Metab. Rev..

[bib0009] Barski O.A., Tipparaju S..M., Bhatnagar A. (2008). The aldo-keto reductase superfamily and its role in drug metabolism and detoxification. Drug Metab. Rev..

[bib0010] Beijer D., Agnew T., Rack J.G.M., Prokhorova E., Deconinck T., Ceulemans B., Peric S., Milic Rasic V., De Jonghe P., Ahel I., Baets J. (2021). Biallelic mutations in complex neuropathy affect ADP ribosylation and DNA damage response. Life Sci. Alliance..

[bib0011] Bhattacharya K., Weidenauer L., Luengo T.M., Pieters E.C., Echeverría P.C., Bernasconi L., Wider D., Sadian Y., Koopman M.B., Villemin M., Bauer C., Rüdiger S.G.D., Quadroni M., Picard D. (2020). The Hsp70-Hsp90 co-chaperone Hop/Stip1 shifts the proteostatic balance from folding towards degradation. Nat. Commun..

[bib0012] Broeks M.H., van Karnebeek C.D.M., Wanders R.J.A., Jans J.J.M., Verhoeven-Duif N.M. (2021). Inborn disorders of the malate aspartate shuttle. J. Inherit. Metab. Dis..

[bib0013] Burillo E., Jorge I., Martínez-López D., Camafeita E., Blanco-Colio L.M., Trevisan-Herraz M., Ezkurdia I., Egido J., Michel J.-B., Meilhac O., Vázquez J., Martin-Ventura J.L. (2016). Quantitative HDL proteomics identifies Peroxiredoxin-6 as a biomarker of human abdominal aortic aneurysm. Sci. Rep..

[bib0014] Calder P.C. (2015). Functional roles of fatty acids and their effects on human health. JPEN J. Parenter. Enteral. Nutr..

[bib0015] Caracciolo G. (2015). Liposome-protein corona in a physiological environment: challenges and opportunities for targeted delivery of nanomedicines. Nanomedicine.

[bib0016] Chanburee S., Tiyaboonchai W. (2017). Mucoadhesive nanostructured lipid carriers (NLCs) as potential carriers for improving oral delivery of curcumin. Drug. Dev. Ind. Pharm..

[bib0017] Chang C., Nickerson M.T. (2018). Stability and in vitro release behaviour of encapsulated omega fatty acid-rich oils in lentil protein isolate-based microcapsules. Int. J. Food Sci. Nutr..

[bib0018] Chen L., Zhao Z.W., Zeng P.H., Zhou Y.J., Yin W.J. (2022). Molecular mechanisms for ABCA1-mediated cholesterol efflux. Cell Cycle.

[bib0019] Colberg L., Cammann C., Greinacher A., Seifert U. (2020). Structure and function of the ubiquitin-proteasome system in platelets. J. Thromb. Haemost..

[bib0020] Cui L., Li H., Xi Y., Hu Q., Liu H., Fan J., Xiang Y., Zhang X., Shui W., Lai Y. (2022). Vesicle trafficking and vesicle fusion: mechanisms, biological functions, and their implications for potential disease therapy. Mol. Biomed..

[bib0021] Dallacasagrande V., Hajjar K.A. (2020). Annexin A2 in inflammation and host defense. Cells.

[bib0022] Defour A., Medikayala S., Van der Meulen J.H., Hogarth M.W., Holdreith N., Malatras A., Duddy W., Boehler J., Nagaraju K., Jaiswal J.K. (2017). Annexin A2 links poor myofiber repair with inflammation and adipogenic replacement of the injured muscle. Hum. Mol. Genet..

[bib0023] Demonbreun A.R., Quattrocelli M.., Barefield D.Y., Allen M.V., Swanson K.E., McNally E.M. (2016). An actin-dependent annexin complex mediates plasma membrane repair in muscle. J. Cell Biol..

[bib0024] Dmitrieff S., Nédélec F. (2016). Amplification of actin polymerization forces. J. Cell Biol..

[bib0025] Drescher D.G., Drescher M..J., Selvakumar D., Annam N.P. (2023). Analysis of Dysferlin direct interactions with putative repair proteins links apoptotic signaling to Ca2+ elevation via PDCD6 and FKBP8. Int. J. Mol. Sci..

[bib0026] Fisher A.B. (2017). Peroxiredoxin 6 in the repair of peroxidized cell membranes and cell signaling. Arch. Biochem. Biophys..

[bib0027] Folch J., Lees M., Sloane Stanley G.H. (1957). A simple method for the isolation and purification of total lipides from animal tissues. J. Biol. Chem..

[bib0028] Foroozandeh P., Aziz A.A. (2018). Insight into cellular uptake and intracellular trafficking of nanoparticles. Nanoscale Res. Lett..

[bib0029] Garibotto G., Picciotto D., Verzola D., Valli A., Sofia A., Costigliolo F., Saio M., Viazzi F., Esposito P. (2023). Homocysteine exchange across skeletal muscle in patients with chronic kidney disease. Physiol. Rep..

[bib0030] Gerling C.J., Mukai K.., Chabowski A., Heigenhauser G.J.F., Holloway G.P., Spriet L.L., Jannas-Vela S. (2019). Incorporation of omega-3 fatty acids into human skeletal muscle sarcolemmal and mitochondrial membranes following 12 weeks of fish oil supplementation. Front. Physiol..

[bib0031] Gonzalez C., Proudfoot M., Brown G., Korniyenko Y., Mori H., Savchenko A., Yakunin A. (2006). Molecular basis of formaldehyde detoxification - characterization of two S-formylglutathione hydrolases from echerichia coli, FrmB and YeiG. J. Biol. Chem..

[bib0032] Hajjar K.A. (2015). The biology of annexin A2: from vascular fibrinolysis to innate immunity. Trans. Am. Clin. Climatol. Assoc..

[bib0033] Hang C., Song Y., Li Y., Zhang S., Chang Y., Bai R., Saleem A., Jiang M., Lu W., Lan F., Cui M. (2021). Knockout of MYOM1 in human cardiomyocytes leads to myocardial atrophy via impairing calcium homeostasis. J. Cell. Mol. Med..

[bib0034] Hang T.T.T., Molee W., Khempaka S. (2018). Linseed oil or tuna oil supplementation in slow-growing chicken diets: can their meat reach the threshold of a “high in n-3 polyunsaturated fatty acids” product?. J. Appl. Poult. Res..

[bib0035] Hargreaves M., Spriet L.L. (2020). Skeletal muscle energy metabolism during exercise. Nat. Metab..

[bib0036] Hitosugi T., Zhou L., Elf S., Fan J., Kang H.B., Seo J.H., Shan C., Dai Q., Zhang L., Xie J., Gu T.L., Jin P., Alečković M., LeRoy G., Kang Y., Sudderth J.A., DeBerardinis R.J., Luan C.H., Chen G.Z., Muller S., Shin D.M., Owonikoko T.K., Lonial S., Arellano M.L., Khoury H.J., Khuri F.R., Lee B.H., Ye K., Boggon T.J., Kang S., He C., Chen J. (2012). Phosphoglycerate mutase 1 coordinates glycolysis and biosynthesis to promote tumor growth. Cancer Cell.

[bib0037] Holness M.J., Sugden M.C. (2003). Regulation of pyruvate dehydrogenase complex activity by reversible phosphorylation. Biochem. Soc. Trans..

[bib0038] Homyok P., Sonsamrong W., Chainet N., Khosinklang W., Kamkaew A., Yata T., Baéza E., Berri C., Molee A., Molee W. (2025). Optimizing n-3 PUFA dietary enrichment in slow-growing Korat chickens using lipid-based nanoparticles: effects on growth performance, carcass traits, meat quality, meat fatty acid composition, and blood biochemical parameters. Poult. Sci..

[bib0039] Huang L., Tepaamorndech S., Kirschke C.P., Newman J.W., Keyes W.R., Pedersen T.L., Dumnil J. (2018). Aberrant fatty acid metabolism in skeletal muscle contributes to insulin resistance in zinc transporter 7 (znt7)-knockout mice. J. Biol. Chem..

[bib0040] Jackson M.J., Pollock N.., Staunton C., Jones S., McArdle A. (2022). Redox control of signalling responses to contractile activity and ageing in skeletal muscle. Cells.

[bib0041] Jaiswal J.K., Lauritzen S..P., Scheffer L., Sakaguchi M., Bunkenborg J., Simon S.M., Kallunki T., Jäättelä M., Nylandsted J. (2014). S100A11 is required for efficient plasma membrane repair and survival of invasive cancer cells. Nat. Commun..

[bib0042] Jannas-Vela S., Espinosa A., Candia A.A., Flores-Opazo M., Peñailillo L., Valenzuela R. (2023). The role of omega-3 polyunsaturated fatty acids and their lipid mediators on skeletal muscle regeneration: a narrative review. Nutrients.

[bib0043] Jones P.D., Ricke S.C. (2021). Challenges in developing nano-delivery systems for enhancing the bioavailability of nutrients and phytochemicals: a review. Crit. Rev. Food Sci. Nutr..

[bib0044] Karan B.M., Little K.., Augustine J., Stitt A.W., Curtis T.M. (2023). Aldehyde dehydrogenase and aldo-keto reductase enzymes: basic concepts and emerging roles in diabetic retinopathy. Antioxidants.

[bib0045] Katsnelson G., Ceddia R.B. (2020). Docosahexaenoic and eicosapentaenoic fatty acids differentially regulate glucose and fatty acid metabolism in L6 rat skeletal muscle cells. Am. J. Physiol. Cell Physiol..

[bib0046] Khosinklang W., Kubota S., Riou C., Keawsatuan P., Molee A., Molee W. (2023). Omega-3 meat enrichment and L-FABP, PPARA and LPL genes expression are modified by the level and period of tuna oil supplementation in slow-growing chickens. J. Anim. Sci..

[bib0047] Krcmery J., Camarata T., Kulisz A., Simon H.G. (2010). Nucleocytoplasmic functions of the PDZ-LIM protein family: new insights into organ development. Bioessays.

[bib0048] Kusunoki C., Yang L., Yoshizaki T., Nakagawa F., Ishikado A., Kondo M., Morino K., Sekine O., Ugi S., Nishio Y., Kashiwagi A., Maegawa H. (2013). Omega-3 polyunsaturated fatty acid has an anti-oxidant effect via the nrf-2/HO-1 pathway in 3T3-L1 adipocytes. Biochem. Biophys. Res. Commun..

[bib0049] Lalia A.Z., Dasari S.., Robinson M.M., Abid H., Morse D.M., Klaus K.A., Lanza I.R. (2017). Influence of omega-3 fatty acids on skeletal muscle protein metabolism and mitochondrial bioenergetics in older adults. Aging (Albany NY).

[bib0050] Lamber E.P., Guicheney P.., Pinotsis N. (2022). The role of the M-band myomesin proteins in muscle integrity and cardiac disease. J. Biomed. Sci..

[bib0051] Laville E., Sayd T., Morzel M., Blinet S., Chambon C., Lepetit J., Renand G., Hocquette J.F. (2009). Proteome changes during meat aging in tough and tender beef suggest the importance of apoptosis and protein solubility for beef aging and tenderization. J. Agric. Food Chem..

[bib0052] Lee L., Karabina A., Broadwell L., Leinwand L. (2019). The ancient sarcomeric myosins found in specialized muscles. Skelet. Muscle..

[bib0053] Lee S.M., Lee S..H., Jung Y., Lee Y., Yoon J.H., Choi J.Y., Hwang C.Y., Son Y.H., Park S.S., Hwang G.S., Lee K.P., Kwon K.S. (2020). FABP3-mediated membrane lipid saturation alters fluidity and induces ER stress in skeletal muscle with aging. Nat. Commun..

[bib0054] Li N., Liu X. (2020). Phosphoglycerate mutase 1: its glycolytic and non-glycolytic roles in tumor malignant behaviors and potential therapeutic significance. Onco Targets Ther.

[bib0055] Li Q., Hoppe T. (2023). Role of amino acid metabolism in mitochondrial homeostasis. Front. Cell Dev. Biol..

[bib0056] Liu B., Zhang M., Tong F., Yang S., Wang H. (2016). Expression patterns and physiological roles of SH3BGR protein family as adaptor proteins. Integr. Cancer Sci. Ther..

[bib0057] Manson A., Winter T., Aukema H.M. (2023). Phospholipase A2 enzymes differently impact PUFA release and oxylipin formation ex vivo in rat hearts. Prostaglandins Leukot. Essent. Fatty Acids..

[bib0058] Metcalfe L.D., Schmitz A..A., Pelka J.R. (1966). Rapid preparation of fatty acid methyl esters from lipids for gas chromatographic analysis. Anal. Chem..

[bib0059] Michalak M. (2024). Calreticulin: endoplasmic reticulum Ca(2+) gatekeeper. J. Cell. Mol. Med..

[bib0060] Mol M., Regazzoni L., Altomare A., Degani G., Carini M., Vistoli G., Aldini G. (2017). Enzymatic and non-enzymatic detoxification of 4-hydroxynonenal: methodological aspects and biological consequences. Free radic. Biol. Med..

[bib0061] Monastyrskaya K., Babiychuk E.B., Draeger A. (2009). The annexins: spatial and temporal coordination of signaling events during cellular stress. Cell. Mol. Life Sci..

[bib0062] Mulens-Arias V. (2021). Dissecting the inorganic nanoparticle-driven interferences on adhesome dynamics. J. Nanotheranostics..

[bib0063] Murgia M., Nogara L., Baraldo M., Reggiani C., Mann M., Schiaffino S. (2021). Protein profile of fiber types in human skeletal muscle: a single-fiber proteomics study. Skelet. Muscle..

[bib0064] Nabuurs C.I., Choe C..U., Veltien A., Kan H.E., van Loon L.J., Rodenburg R.J., Matschke J., Wieringa B., Kemp G.J., Isbrandt D., Heerschap A. (2013). Disturbed energy metabolism and muscular dystrophy caused by pure creatine deficiency are reversible by creatine intake. J. Physiol..

[bib0065] Nemoto T.K., Fukuma Y.., Itoh H., Takagi T., Ono T. (2006). A disulfide bridge mediated by cysteine 574 is formed in the dimer of the 70-kDa heat shock protein. J. Biochem..

[bib0066] Penning T.M. (2015). The Aldo-keto reductases (AKRs): overview. Chem. Biol. Interact..

[bib0067] Prill K., Carlisle C., Stannard M., Windsor Reid P.J., Pilgrim D.B. (2019). Myomesin is part of an integrity pathway that responds to sarcomere damage and disease. PLOS ONE.

[bib0068] Prislusky M.I., Lam J.G.T., Contreras V.R., Ng M., Chamberlain M., Pathak-Sharma S., Fields M., Zhang X., Amer A.O., Seveau S. (2024). The septin cytoskeleton is required for plasma membrane repair. EMBO Rep.

[bib0069] Qin M., Zhang J., Li M., Yang D., Liu D., Song S., Fu J., Zhang H., Dai W., Wang X., Wang Y., He B., Zhang Q. (2020). Proteomic analysis of intracellular protein corona of nanoparticles elucidates nano-trafficking network and nano-bio interactions. Theranostics.

[bib92] R Core Team (2024). R: A Language and Environment for Statistical Computing.

[bib0070] Schulze A., Tran C., Levandovskiy V., Patel V., Cortez M. (2016). Systemic availability of guanidinoacetate affects GABAA receptor function and seizure threshold in GAMT deficient mice. Amino Acids.

[bib0071] Simopoulos A.P. (2008). The importance of the omega-6/omega-3 fatty acid ratio in cardiovascular disease and other chronic diseases. Exp. Biol. Med. (Maywood)..

[bib0072] Singhal S.S., Singh S..P., Singhal P., Horne D., Singhal J., Awasthi S. (2015). Antioxidant role of glutathione S-transferases: 4-hydroxynonenal, a key molecule in stress-mediated signaling. Toxicol. Appl. Pharmacol..

[bib0073] Smith B., Adams H., Doe Q. (2020). Innovations in nano-delivery systems for enhancing bioavailability of omega-3 rich oils: applications in food and nutrition. Int. J. Nanomedicine..

[bib0074] Stull J.T., Kamm K.E., Vandenboom R. (2011). Myosin light chain kinase and the role of myosin light chain phosphorylation in skeletal muscle. Arch. Biochem. Biophys..

[bib0075] Suzuki R., Nagata K., Yumoto F., Kawakami M., Nemoto N., Furutani M., Adachi K., Maruyama T., Tanokura M. (2003). Three-dimensional solution structure of an archaeal FKBP with a dual function of peptidyl prolyl cis-trans isomerase and chaperone-like activities. J. Mol. Biol..

[bib0076] Syed N.A., Bhatti A.., John P. (2023). Molecular link between glo-1 expression and markers of hyperglycemia and oxidative stress in vascular complications of type 2 diabetes mellitus. Antioxidants (Basel).

[bib0077] Tachtsis B., Camera D., Lacham-Kaplan O. (2018). Potential roles of n-3 PUFAs during skeletal muscle growth and regeneration. Nutrients.

[bib0078] Tian C., Asghar S., Wu Y., Chen Z., Jin X., Yin L., Huang L., Ping Q., Xiao Y. (2017). Improving intestinal absorption and oral bioavailability of curcumin via taurocholic acid-modified nanostructured lipid carriers. Int. J. Nanomedicine..

[bib0079] Tomczyk M. (2024). Omega-3 fatty acids and muscle strength—current state of knowledge and future perspectives. Nutrients.

[bib0080] Vistoli G., De Maddis D., Cipak A., Zarkovic N., Carini M., Aldini G. (2013). Advanced glycoxidation and lipoxidation end products (AGEs and ALEs): an overview of their mechanisms of formation. Free Radic. Res..

[bib0081] Wang H., Zhou R., Sun L., Xia J., Yang X., Pan C., Huang N., Shi M., Bin J., Liao Y., Liao W. (2017). TOP1MT deficiency promotes GC invasion and migration via the enhancements of LDHA expression and aerobic glycolysis. Endocr. Relat. Cancer.

[bib0082] Wang H., Zhang Z., Guan J., Lu W., Zhan C. (2021). Unraveling GLUT-mediated transcytosis pathway of glycosylated nanodisks. Asian J. Pharm. Sci..

[bib0083] Wang R., He J., Xu Y., Peng B. (2024). Impact of protein coronas on lipid nanoparticle uptake and endocytic pathways in cells. Molecules.

[bib0084] Wangngae S., Chansaenpak K., Yostawonkul J., Mahingsadet K., Jinaphon T., Molee W., Kamkaew A. (2023). Lipid-based nanoparticles as targeted delivery system in Korat chicken. ACS Food Sci. Technol..

[bib0085] Yang X., Song J., Yan L.J. (2019). Chronic inhibition of mitochondrial dihydrolipoamide dehydrogenase (DLDH) as an approach to managing diabetic oxidative stress. Antioxidants (Basel).

[bib0086] Yeh Y.C., Kim S..T., Tang R., Yan B., Rotello V.M. (2014). Insulin-based regulation of glucose-functionalized nanoparticle uptake in muscle cells. J. Mater. Chem. B..

[bib0087] Yin H., Zhao J., He H., Chen Y., Wang Y., Li D., Zhu Q. (2020). Gga-miR-3525 targets PDLIM3 through the MAPK signaling pathway to regulate the proliferation and differentiation of skeletal muscle satellite cells. Int. J. Mol. Sci..

[bib0088] Zhang S., Cai Z., Li H. (2023). AHNAKs roles in physiology and malignant tumors. Front. Oncol..

[bib0089] Zhang Z., Zhang L., Zhou L., Lei Y., Zhang Y., Huang C. (2019). Redox signaling and unfolded protein response coordinate cell fate decisions under ER stress. Redox Biol.

[bib0090] Zhao J.M., Qi T.G. (2021). The role of TXNL1 in disease: treatment strategies for cancer and diseases with oxidative stress. Mol. Biol. Rep..

[bib0091] Zhen Y., Radulovic M., Vietri M., Stenmark H. (2021). Sealing holes in cellular membranes. EMBO J.

